# Using Feature Engineering and Principal Component Analysis for Monitoring Spindle Speed Change Based on Kullback–Leibler Divergence with a Gaussian Mixture Model

**DOI:** 10.3390/s23136174

**Published:** 2023-07-05

**Authors:** Yi-Cheng Huang, Ching-Chen Hou

**Affiliations:** Department of Mechanical Engineering, National Chung Hsing University, Taichung 402, Taiwan; hcc106822@gmail.com

**Keywords:** machine tool, Gaussian mixture model, principal component analysis, feature engineering

## Abstract

Machining is a crucial constituent of the manufacturing industry, which has begun to transition from precision machinery to smart machinery. Particularly, the introduction of artificial intelligence into computer numerically controlled (CNC) machine tools will enable machine tools to self-diagnose during operation, improving the quality of finished products. In this study, feature engineering and principal component analysis were combined with the online and real-time Gaussian mixture model (GMM) based on the Kullback–Leibler divergence’s measure to achieve the real-time monitoring of changes in manufacturing parameters. Based on the attached accelerometer device’s vibration signals and current sensing of the spindle, the developed GMM unsupervised learning was successfully used to diagnose the spindle speed changes of a CNC machine tool during milling. The F1-scores with improved experimental results for X, Y, and Z axes were 0.95, 0.88, and 0.93, respectively. The established FE-PCA-GMM/KLD method can be applied to issue warnings when it predicts a change in the manufacturing process parameter. A smart sensing device for diagnosing the machining status can be fabricated for implementation. The effectiveness of the developed method for determining the manufacturing parameter changes was successfully verified by experiments.

## 1. Introduction

In the manufacturing industry, every aspect of a machine tool directly affects the quality of the finished product in terms of geometric precision, surface roughness, or other criteria. From the perspectives of a good finished product and determining the machine tool status, such as part wear or other abnormalities, the machine operation was previously handled by on-site engineers. However, this method requires high staffing costs and cannot guarantee a consistent finished product quality. Accordingly, the objectives of new smart manufacturing methods are to reduce time costs and improve product quality. Some scholars have attempted to improve product quality by optimizing controller, finishing, or interpolation parameters. For instance, Chiu and Lee [1] proposed an intelligent machining system named the adaptive-network-based fuzzy inference system (ANFIS) that was optimized using particle swarm optimization. Selecting optimal computer numerical control (CNC) controller parameters was a key factor for obtaining favorable performance indicators because these five parameters, namely jerk, acceleration, feed rate, angular jerk, and centripetal acceleration, affect the interpolation process of both trajectory and speed planning. After analyzing the machining data to obtain contouring and tracking errors, Chiu and Lee used ANFIS to establish a relationship model between the five aforementioned CNC parameters and three machining performance indices, namely, speed, milling accuracy, and surface smoothness. Hence, users could adjust the weight of each performance index in accordance with their needs, and ANFIS could generate the optimal CNC parameters accordingly. Tsai et al. [2] proposed an optimization algorithm that used a backpropagation neural network to predict three machining performance indices based on interpolated parameters (acceleration, time constant, S-shaped time constant, corner velocity, and feed rate). These parameters were treated as the input. Then, they applied the genetic algorithm (GA) to search for the optimal interpolation parameters based on an objective function without constraints. The accuracy and efficiency of this algorithm were verified by the study results. Huang and Liao [3] used a GA-optimized general regression neural network to build a model that predicts the corner error, 45° corner jerk, 60° corner jerk, and machining time from CNC controller parameters. And they successfully established a system that generates a set of optimized CNC parameters in accordance with user-provided machining requirements. However, these three studies performed parameter optimization for machine tools in healthy or normal states. They did not discuss parameter optimization for machine tools with deteriorated components, which may lead to inconsistencies between the machine tool state and the machining, interpolation, or controller parameter values.

From the perspective of machine health status, machine tools state monitoring is mainly performed using supervised learning. Azamfar et al. [4] proposed a deep-learning-based domain adaptation method that used a deep convolutional neural network to perform feature extraction and identify machine health status. They used the maximum mean discrepancy to evaluate and optimize data distributions in various operating conditions and validated the method by monitoring a ball screw in different conditions. The results indicated that their proposed method can effectively extract universal features for fault diagnosis. Lin et al. [5] proposed an innovative smart system for the early detection of failures in automatic tool change (ATC) systems. The open and close signals of the system’s tool magazine door were used as input, and 41 indicators were obtained from 26 machines with statistics-based feature extraction methods. These were used to train lightweight supervised learning algorithms, such as support vector machine and k-nearest neighbor, for early failure detection. Their system classified the ATC system state into predefined risk levels of 0 for “normal,” 1 for “caution,” and 2 for “danger.” They further introduced feature selection, correlation, and regression models to assist in the extraction of features associated with ATC failure. Their smart box can monitor ATC system states in real time on the production line and discover potential failures in advance. Li et al. [6] developed an approach to monitoring the health conditions of cutting tools in CNC machine tools. A high-precision Hall sensor was used to collect spindle current data from the CNC. These data can reflect the tool state, such as the cutting force, tool wear, and tool breakage, during machining. A deep learning model for anomaly detection was constructed by analyzing features such as the oscillations and frequencies in the spindle current signal. This model used a simple and deep learning model denoted CNN-AD to learn and extract features in the spindle current signal for abnormality detection. The effectiveness of the model was validated by testing on an experimental dataset that included signals generated both during normal operation and anomalous operation, such as with tool wear or tool breakage. The results demonstrated that their proposed model could accurately detect tool abnormalities. Irgat et al. [7] collected three-axis vibration signals of a healthy motor and a motor with inner-race and outer-race bearing faults. They found that peak-to-peak and root-mean-square statistical features for each vibration axis are the key features for differentiating between healthy and faulty motors. Classification was performed using the k-nearest-neighbor algorithm and decision trees because of their relative simplicity, since k-nearest neighbor is known for its simplicity and low computational complexity. And the decision tree has low computational costs, high efficiency, and high speed. Ball screws are widely used in mechanical processes, but long operation leads to reductions in the preload and rigidity of the lead screw, resulting in inaccurate positioning. However, these supervised learning approaches are not without shortcomings—the data must be labeled, which requires high labor costs [8]. For a supervised-learning tool wear model [9] to determine the tool wear value or lifespan for a tooling process, it must be provided with a dataset labeled with these values for the specific tooling conditions of that process. Any changes to the tool type, workpiece material, or cutting parameters may reduce model accuracy, restricting the practical applications of state diagnosis methods.

Hence, researchers have begun investigating unsupervised learning for failure detection. Hong et al. [10] presented an effective analytical technique for the early diagnosis of ball bearing faults based on vibration signals from bearings by using the feature extraction technique based on spectral kurtosis (SK) and a Gaussian mixture model (GMM) to evaluate bearing defects. Principal component analysis (PCA) was employed to reduce misclassifications due to noise. The SK and root-mean-square (RMS) diagnostic results for an experiment were compared, and the SK-GMM results could effectively evaluate the level and severity of faults. The use of the extracted features, PCA, and GMM for composite structures was shown in [11]. Guo and Chen [12] developed a variable-refrigerant-flow air-conditioning system by modifying the PCA-GMM fault diagnosis model and rating four types of system faults. The experimental results indicated that incorporating PCA increased the fault diagnosis accuracy and reduced runtime. Yu [13] used PCA and GMM by solving the nonlinearity and multimodal trajectories issues for the semiconductor manufacturing system. Liu et al. [14] proposed a GMM-based outlier detection algorithm, which used a global optimization expectation maximization algorithm to fit the testing dataset to a GMM. They adopted the Mahalanobis squared distance as the measure index. For each Gaussian component, data points more than three standard deviations from the mean were considered outliers. Experiments involving the simulation dataset and the real dataset verified the effectiveness of the algorithm in detecting outliers in high-dimensional datasets. In [15], a Hellinger distance measure with GMM was discussed for bearing prognosis. Nam and Kwon [16] developed a model that combined an LSTM-Autoencoder for feature extraction and GMM to create a precise model of tool breakage. Combining the approaches improved the detection accuracy and response speed. He et al. [17] developed a fast clustering algorithm and GMM for the fault prognosis of a photovoltaic inverter. Lucà et al. [18] proposed a GMM-based damage detection approach for axially loaded beam-like structures. After damaging a specific part of the test structure, a sensor was used to measure the vibration response. And GMM was analyzed and was used to model the response signal. The eigenfrequencies of multiple vibration modes were composed into a multivariate damage features. By framing the damage detection problem as an unsupervised outlier detection problem and using the Mahalanobis squared distance to define the effective damage index, this proposed approach could accurately detect the location and degree of the damage with higher precision and sensitivity than existing methods. In [19], to avoid hidden misclassification for the bearing fault diagnosis of industrial electric motor, a broadened GMM-window-based signal processing approach was proposed. Zhang [20] et al. studied an improved ensemble empirical mode decomposition hard threshold denoising (EEMD-HTD) and GMM multisource sensor fault diagnosis method for chillers. EEMD-HTD decomposed data signals through empirical mode decomposition and then input the decomposed signals to the GMM model to detect and diagnose chiller sensor faults. They used their approach to detect faults in a heating, ventilation, and air conditioning (HVAC) chiller system and validated the effectiveness of their approach in diagnosing single-source and multisource HVAC faults. Some useful EMD information of intrinsic model functions gathered with PCA and GMM for the pump’s degradation was studied by [21]. Jianbo Yu [22] presented an adaptive GMM (AGMM) and employed Kullback–Leibler divergence (KLD) as an indicator for quantifying tool performance degradation. AGMM is capable of the online and adaptive learning of the dynamic changes in tool performance throughout the tool lifetime by dynamically adjusting the learning rate and parameters and merging and splitting Gaussian components. Moreover, tool performance changes were quantified by measuring the distance between density distributions produced by the AGMM and the baseline GMM. After conducting a machine tool test, the experimental results demonstrated that the AGMM-based KLD indicator is effective for assessing tool performance degradation. In [23], the PCA and KLD were used as a method for improving an incipient fault detection of an electrical drive system under the multivariate statistical analysis frame. Cao et al. [24] proposed a GMM-based variational Bayesian PCA with KLD for the industrial hydrocracking process. They succeeded in detecting the incipient faults without monitoring delay.

As mentioned, in CNC machine tools diagnostic studies, the tool type, workpiece material, and cutting parameters affect the results of supervised learning when determining tool wear during the machining process. Parameter combinations in actual machining change frequently. Previous studies have not investigated some potential anomalies, for example, reducing spindle speed as a protective measure when encountering cutting resistance. This may result in gaps in the cutting surface that affect the finish quality. Some surface anomalies or poor spindle temperature control may also cause the spindle speed change or lead to spindle displacement. Restrictions on the CNC feed axis servo acceleration and jerk settings can cause stress changes on the tool surface at the moment of feed. The supervised learning and monitoring of these scenarios during actual machining states are difficult. Clustering algorithms, such as k-means or hierarchical clustering, only yield the center point and size of each cluster. They do not describe the data distribution or trends within each cluster. Therefore, GMM can estimate the parameters of each Gaussian distribution (i.e., the means and covariance matrix) and thereby determine the probability density function of the whole dataset, offering a richer description of the data.

The static and dynamic forces in between the spindle tool and workpiece are extremely complicated when machining a product. A smart spindle is mostly fabricated and embedded with an accelerator and a temperature sensor in smart manufacturing. However, if it is not a smart spindle, an attached accelerator near to the spindle is a solution to the sensing source for diagnosis. A dynameter under the workpiece for determining the dynamic force of the spindle tool is also used. However, the dynameter costs a lot and is not very practical in industrial applications. In [25], the cutting path effect on the acoustic emission (AE) and vibration signals during the micro-milling processes was investigated. Their AE sensor and an accelerometer were installed on a fixture attached to the spindle housing. An acoustic emission (AE) sensor attached to the device for the diagnosis of the tool wear can be used. However, it is not easy to explain the phenomena of the deformed metal lattices signals when the spindle’s cutting speed is changed. So, in this study, we adopted a practical approach for industry application. An accelerator was directly attached to the device. This will be a new solution for embedding a MEMS accelerator or an add-on accelerator for a smart device of CNC machine tools. As such, in this study, GMM is combined with feature engineering, PCA, and KLD to establish an unsupervised learning approach. A diagnostic model for detecting the spindle speed change during surface milling was implemented.

## 2. Research Methods

### 2.1. Experimental Machine

**Figure 1:**
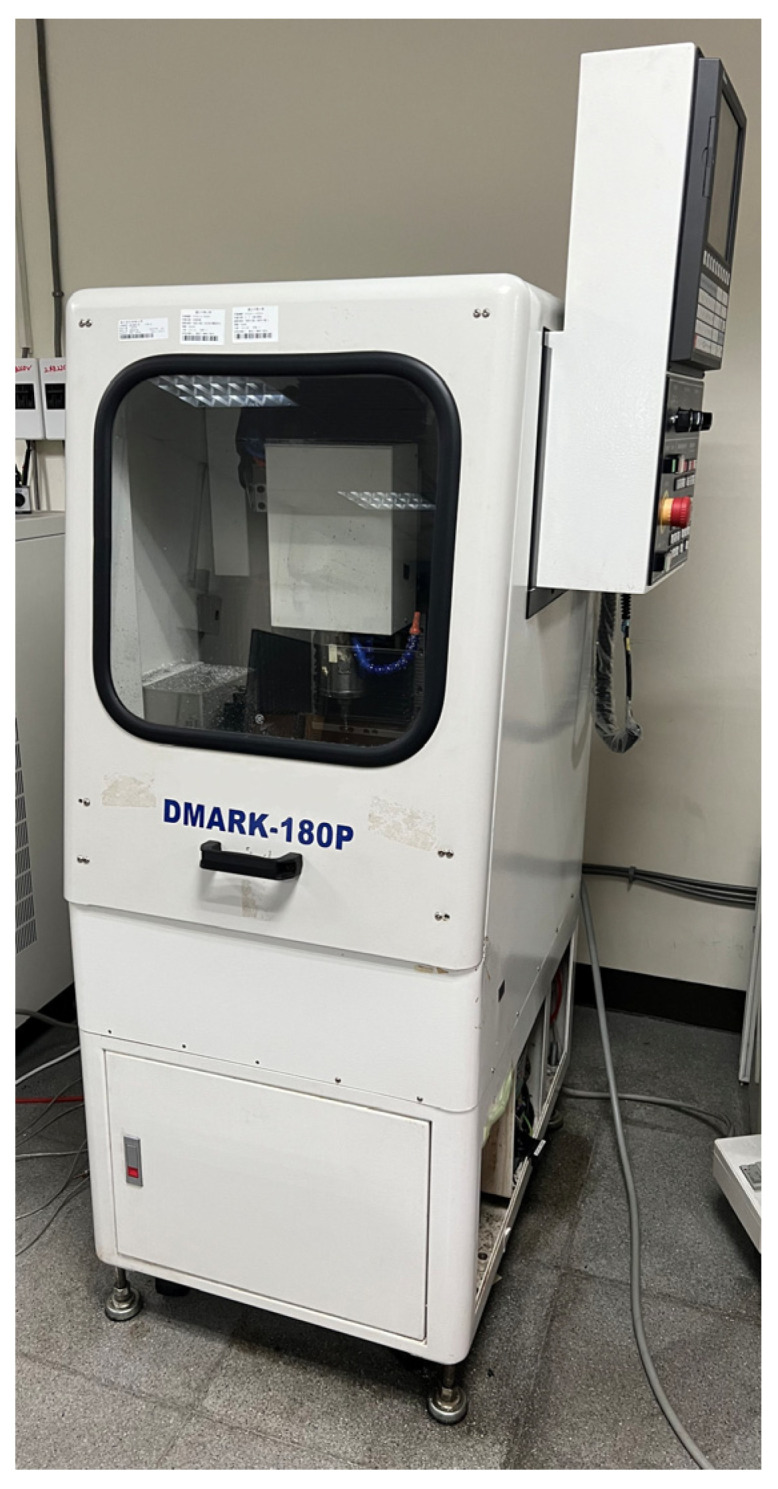
DMARK-180P by Dmark.

**Figure 2:**
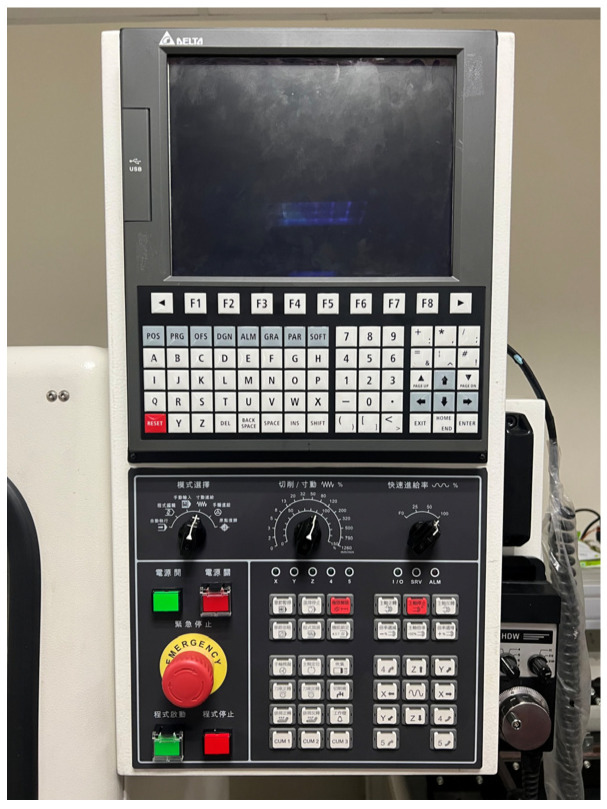
Delta NC311A-MS-A CNC.

**Figure 3:**
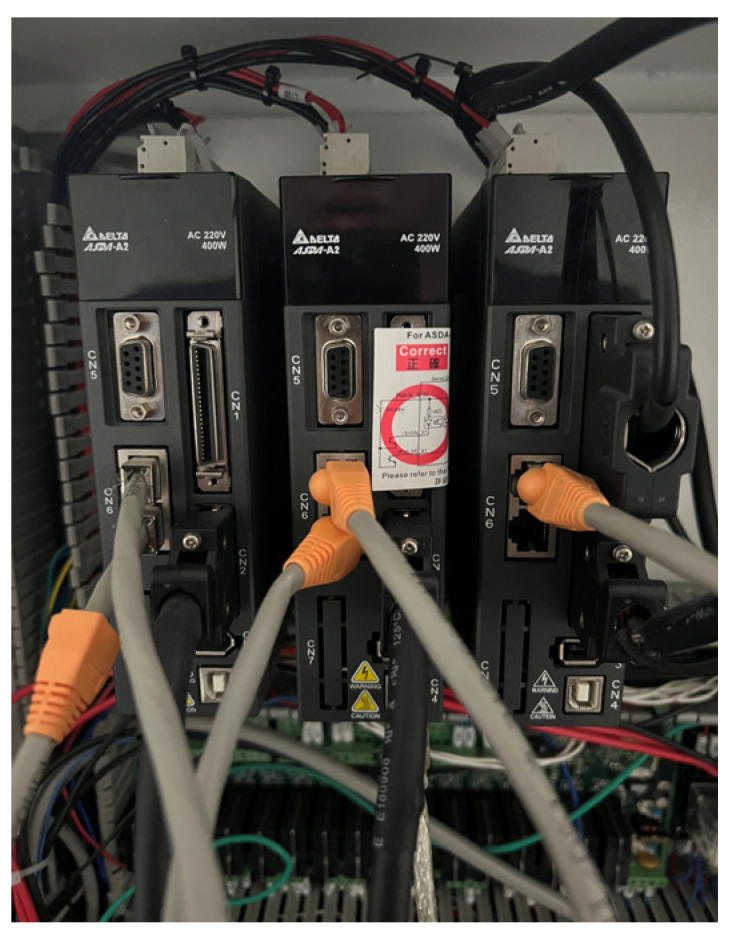
Delta ASDA-A2 servo drive.

The CNC tooling machine used in this study was the DMARK-180P (Figure 1, Dmark Co., Taichung, Taiwan), which was equipped with a Delta NC31A-MS-A CNC (Figure 2, Delta Electronics, Taipei, Taiwan) and AC 220-V 400-W servo drive from the ASDA-A2 series (Figure 3, Delta Electronics). The specifications of the machine are presented in Table 1.

### 2.2. DAQ-204 Vibration-Capture Module and Triaxial Accelerometer

**Figure 4:**
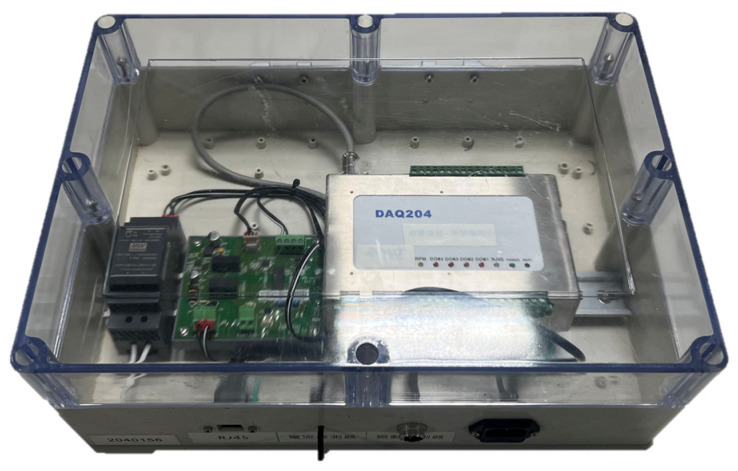
BALTech DAQ-204 vibration module.

**Figure 5:**
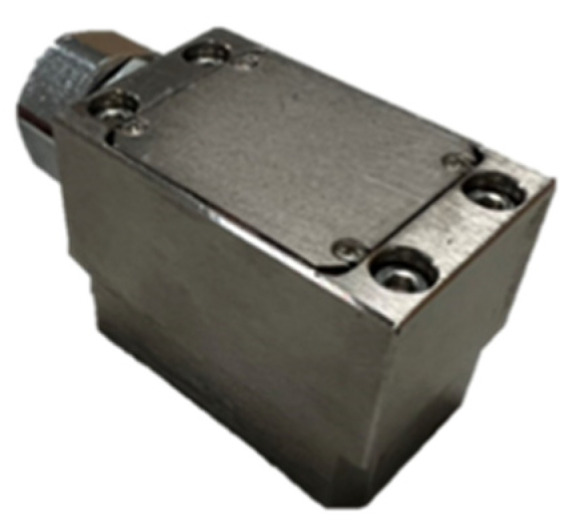
Picture of the BT-1513 triaxial accelerometer.

The BALTech DAQ-204 vibration module (Figure 4, Bal Tech Co., Hsinchu, Taiwan) was primarily used to detect vibrations and record raw vibration data. Its product specifications are listed in Table 2. The accelerometer used in this study was the BT-1513 triaxial accelerometer (Figure 5). Its sampling frequency was set to 12,800 Hz, and gravitational acceleration (g) was the sampling unit. Its specifications are listed in Table 3.

### 2.3. Gaussian Mixture Model

GMM is a mixture model that is widely used in unsupervised data grouping and has been successfully applied to voice and speech recognition. GMM uses multiple Gaussian distributions to represent the distribution of eigenvectors. Due to GMM’s ability to record the categories and positions demonstrated in the data and also to describe the size and shape of these categories in space, it is suitable for producing color visualizations of the eigenvector distribution in space.

A GMM has three parameters: the mixture weight, the mean vector, and the covariance matrix. These parameters were gathered and given new symbols, as follows:

(1) $$(a_{i},\mu_{i},\Sigma_{i}),i=1,2,...,V$$

where $a_{i}$ is the mixture weight, $\mu_{i}$ is the mean vector, $\Sigma_{i}$ is the covariance matrix, and V is the number of Gaussian distributions. If a set of points in *d*-dimensional space can be described as $x_{n}$, *n* = 1 … *N*, the Gaussian frequency function $g\left(x_{n};\mu_{i},\Sigma_{i}\right)$ can be used to describe the probability density function of the points [26]:

(2) $$g\left(x_{n};\mu_{i},\Sigma_{i}\right)=\frac{1}{\sqrt{{\left(2\pi \right)}^{d}\left|\Sigma_{i}\right|}}exp\left[-\frac{1}{2}{\left(x_{n}-\mu_{i}\right)}^{T}{\Sigma_{i}}^{-1}\left(x_{n}-\mu_{i}\right)\right]$$

For this Gaussian density function, if $x_{i}$, *i* = 1 … *N* are assumed to be mutually exclusive events, then the probability density of the classification clustering that can satisfy the sum *N* of Equation (2) is

(3) $$p\left(x_{n};\mu_{i},\Sigma_{i}\right)=\prod_{i=1}^{n}g\left(x_{n};\mu_{i},\Sigma_{i}\right)$$

where $g\left(x_{n};\mu_{i},\Sigma_{i}\right)$ is the probability density.

An iterative expectation maximization (EM) algorithm is typically used to solve for the GMM parameters. The first step is to calculate the expectation values; this is called the E step. The second step is the maximization step or M step. The maximum likelihood value obtained in the E step is used to calculate the parameter value, and the parameter estimates in the M step are used for the calculations of the E step in the subsequent iteration. In GMM, the EM algorithm can be expressed as follows:

(1) Expectation Step

(4) $$\omega_{i}^{\left(t\right)}=\frac{a_{i}^{\left(t\right)}g\left(x_{n}|\mu_{i}^{\left(t\right)},\Sigma_{i}^{\left(t\right)}\right)}{\sum_{j=1}^{N}a_{j}^{\left(t\right)}g\left(x_{n}|\mu_{j}^{\left(t\right)},\Sigma_{j}^{\left(t\right)}\right)},\forall n,i$$

where $\omega_{i}^{\left(t\right)}$ is the posterior probability function of the *i*th distribution and $a_{i}^{\left(t\right)}$ is equal to the weight of each Gaussian distribution between 0 and 1.

(2) Expectation Step

(5) $$h_{i}^{\left(t\right)}=\sum_{j=1}^{N}\omega_{j}^{\left(t\right)}\left(x_{n}\right)$$

(6) $$a_{i}^{\left(t+1\right)}=\frac{h_{i}^{\left(t\right)}}{N}$$

(7) $$u_{i}^{\left(t+1\right)}=\frac{1}{h_{i}^{\left(t\right)}}\sum_{j=1}^{N}\omega_{i}^{\left(t\right)}x_{j}$$

(8) $$\Sigma_{i}^{\left(t+1\right)}=\frac{1}{h_{i}^{\left(t\right)}}\Sigma_{j=1}^{N}\Sigma_{i=1}^{V}\omega_{j}^{\left(t\right)}\left(x_{j}-\mu_{i}^{\left(t+1\right)}\right){\left(x_{j}-u_{i}^{\left(t+1\right)}\right)}^{T}$$

where $a_{i}^{\left(t+1\right)}$ is the updated mixture weight, $u_{i}^{\left(t+1\right)}$ is the updated mean vector, and $\Sigma_{i}^{\left(t+1\right)}$ is the updated covariance matrix.

### 2.4. Feature Engineering

Raw data typically require preprocessing to extract features before being input to a machine learning model. This process is called feature engineering and aims to extract useful features, remove redundant features, and convert the data into a form that can be used by machine learning algorithms. Feature engineering techniques that can yield effective feature data include feature selection, feature extraction, and outlier reduction.

#### 2.4.1. Feature Extraction

Feature extraction is the expansion of one-dimensional data into numerous dimensions by calculating various statistical indicators, such as by calculating the RMS, skewness, kurtosis, crest factor, variance, and mean of time-domain data or determining the power spectral density of frequency-domain data. After magnifying one-dimensional data into multidimensional data, feature selection can be performed to identify useful feature patterns.

#### 2.4.2. Outlier Reduction

Outliers in the feature extraction results must be removed to prevent their interference with the training model. A common practice is using the *Z* score. Each value is scored by its distance from the mean in terms of the standard deviation, and variables with scores outside of a given range are considered outliers and are removed. In this study, the Z score threshold was 4; that is, values greater than 4 standard deviations from the mean were considered outliers. The Z score of a value is calculated as follows:

(9) $$Z\;Score=\frac{x_{i}-\mu }{\sigma }.$$

where $x_{i}$ is any datum among the 𝑛 data, 𝜇 is the mean of these 𝑛 data, and 𝜎 is the standard deviation.

#### 2.4.3. Feature Selection

In 1933, Harold Hotelling [27] developed PCA, a commonly used approach for reducing the dimensionality of a dataset. It converts high-dimensional data into low-dimensional data through linear transformations while maximizing the retained information. The basis of this conversion is identifying a set of principal components that are mutually orthogonal and maximizing the variance of the data. Selecting the principal components before building a GMM can remove noise, reduce the complexity of the model, and accelerate training.

The concrete steps of feature extraction in PCA are as follows:

(1) Standardize the raw data: Assuming that the raw data are represented by $x_{n}$, *n* = 1 … *N*, the *Z* score can be described as $z_{i}\left(x_{n};\mu_{i},\sigma_{i}\right)$.

(10) $$z_{i}\left(x_{n};\;\mu_{i},\;\sigma_{i}\right)=\frac{x_{n}-\mu_{i}\;}{\sigma_{i}},\;i\;=\;1,2,\ldots ,V$$

where $\mu_{i}$ is the mean of $x_{n}$ and $\sigma_{i}$ is the standard deviation.

(2) Calculate the covariance matrix: If *cov*(*x*, *y*) is calculated using the feature training dataset $z_{i}$, *i* = 1, 2, …, *V*, the covariance matrix equation is then

(11) $$cov(x,\;y)=\frac{1}{n-1}\sum_{j=1}^{N}\left(x_{j}-\mu_{x}\right)\left(y_{j}-\mu_{y}\right),n=N$$

(3) Calculate the eigenvalues and eigenvectors of the covariance matrix: in order of greatest to smallest, the eigenvalues of the covariance matrix are $\lambda_{i}$, *i* = 1 … *n*, and the corresponding eigenvectors are $a_{i}$, *i* = 1… *n*.

(4) Calculate the variance cumulative contribution ratio (α) of the first k principal components greater than 0.97:

(12) $$\frac{\sum_{i=1}^{k}\lambda_{i}}{\sum_{j=1}^{46}\lambda_{j}}\geq 0.97$$

(5) Generate the new principal components matrix with dimensionality *k*:

(13) $$X^{*}={\left[a_{1},a_{2},\ldots ,a_{k}\right]}^{T}X^{T}$$

### 2.5. Kullback–Leibler Divergence

Kullback–Leibler divergence (KLD) is also referred to as relative entropy and can be used to analyze the differences between data with different probability density functions (PDFs). KLD is extremely sensitive to small deviations and is therefore effective for early failure diagnosis [26]. In this paper, normal conditions (spindle speed 6000 RPM) and test conditions (spindle speed 5500 RPM) were modeled using GMM to determine the corresponding Gaussian distributions. The baseline distribution was expressed as $p_{A}\left(x\right)$ and the test distribution was $p_{B}\left(x\right)$. The difference between the two Gaussian distributions was analyzed with the KLD.

(14) $$\left.{KL(p}_{A}\left(x\right)\right\|p_{B}\left(x\right))=[log\frac{\left|\;\sum_{B}\;\right|}{\;\sum_{A}\;}]+\mathrm{Tr}(\sum_{B}^{-1}\sum_{A}^{\;})+{\left(\mu_{A}-\mu_{B}\right)}^{T}\sum_{B}^{-1}(\mu_{A}-\mu_{B})-N$$

where *x* is a vector and $\mu_{A}$ and $\mu_{B}$ are two mean values for the distributions $p_{A}\left(x\right)$ and $p_{B}\left(x\right)$, respectively. Tr($\sum_{B}^{-1}\sum_{A}^{\;})$ is the trace of matrix $\sum_{B}^{-1}\sum_{A}^{\;}\;$. *N* is the dimensionality of vector *x*.

### 2.6. F-Sscore

As in statistical analysis of binary classification, F-score is a measure of a test’s accuracy. This is suitable to apply in this study. It is calculated from the precision and recall of the test. Precision is the number of true positive results divided by the number of all positive results, including those not identified correctly. Recall is the number of true positive results divided by the number of all samples that should have been identified as positive. Precision is also known as the positive predictive value, as shown in Equation (15). Recall is also known as the sensitivity in diagnostic binary classification, as shown in Equation (16). The highest possible value of F-score is 1.0, indicating perfect precision and recall. And the lowest possible value is 0 if either precision or recall is zero. In this study, the $F_{1}$ score, as shown in Equation (17), was adopted for the harmonic mean of precision and recall. Therefore, it represented both precision and recall in one metric for diagnosing the state of spindle speed when it was changed to another unwanted rpm. The range of $F_{1}$ is in between 0 and 1. The 1 represents the best diagnosed performance.

(15) $$precision=\frac{TP}{TP+FP}$$

(16) $$recall=\frac{TP}{TP+FN}$$

(17) $$F_{1}=2\times \frac{precision\times recall}{precision+recall}$$

## 3. Experiments

**Figure 6:**
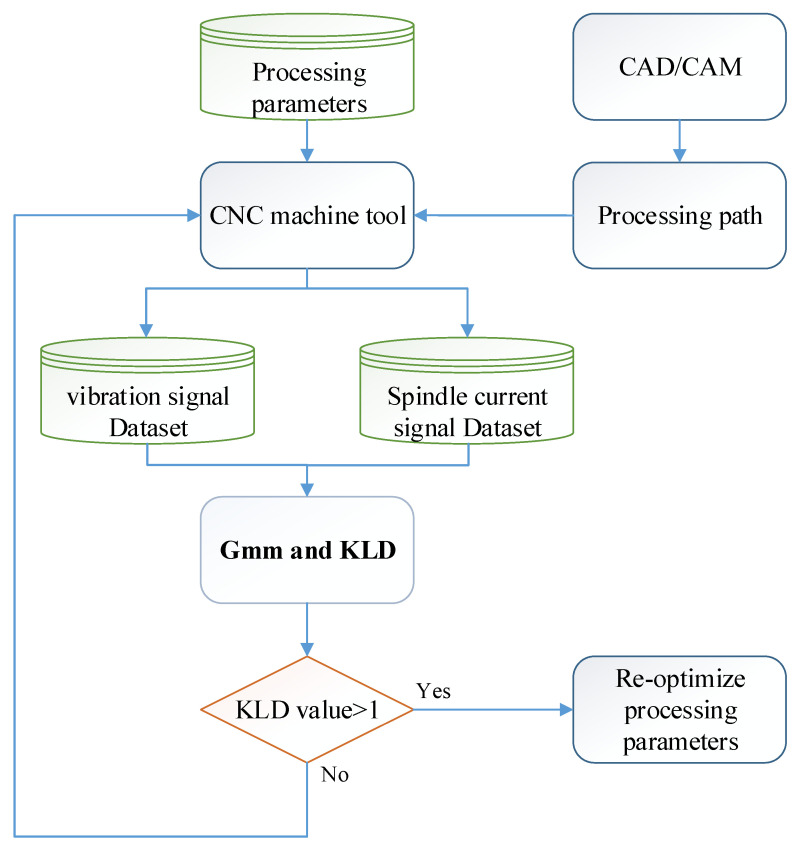
Experimental schema.

**Figure 7:**
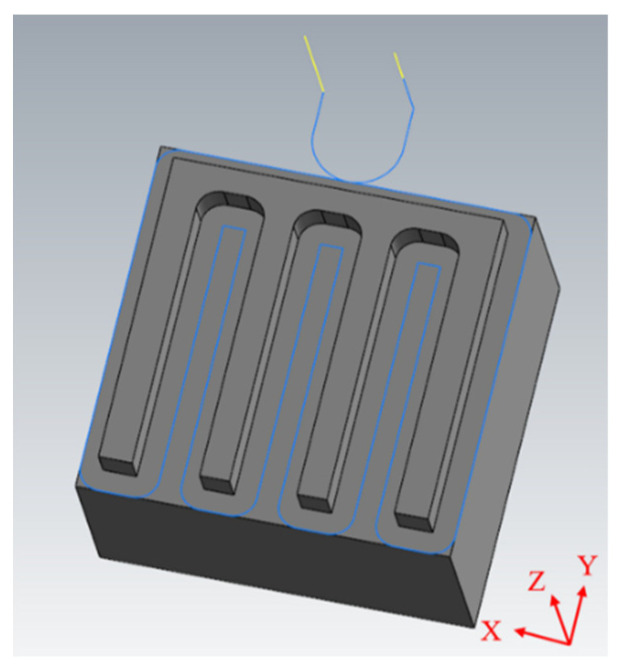
Experimental machining path (blue line is with the G01, G02, G03 et al. NC codes, the yellow lines is with the G00 NC code).

**Figure 8:**
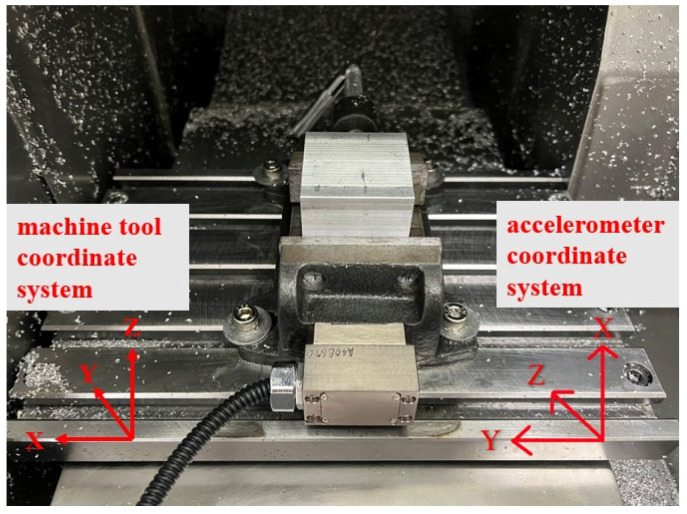
Picture of the accelerometer position attached to the workpiece.

The machined workpiece performance indices for CNC manufacturing depend on the requirement of less machining time, less surface roughness, and less geometric tolerances. In manufacturing, senior engineers set appropriate cutting parameters (such as restrictions on the feed axis acceleration and jerk, the feed rate, and the cutting depth) in accordance with the tooling requirements and the condition of each CNC machine. The experimental schema for process identification of spindle speed under abnormal input cutting parameters is presented in Figure 6. In each experiment, the test workpiece was machined along a single path (Figure 7) and a corner turn was followed by another straight line by a cutting depth of 1 mm eight times. The initial half cut was made with a spindle speed of 6000 RPM. In the rest of the half cut of the following path, the spindle speed was reduced to 5500 RPM by mimicking a tooling parameter abnormality. An accelerometer was affixed to the vice to collect data during the tooling process. A picture of the accelerometer position attached to the workpiece is shown in Figure 8, with the coordinates demonstrated in the bottom-right corner. GMM with real-time monitoring was then used to analyze the differences between the normal and abnormal cutting parameters during milling. In practice, the differences beyond a threshold will prompt an alert in the man–machine interface for the user.

**Figure 9:**
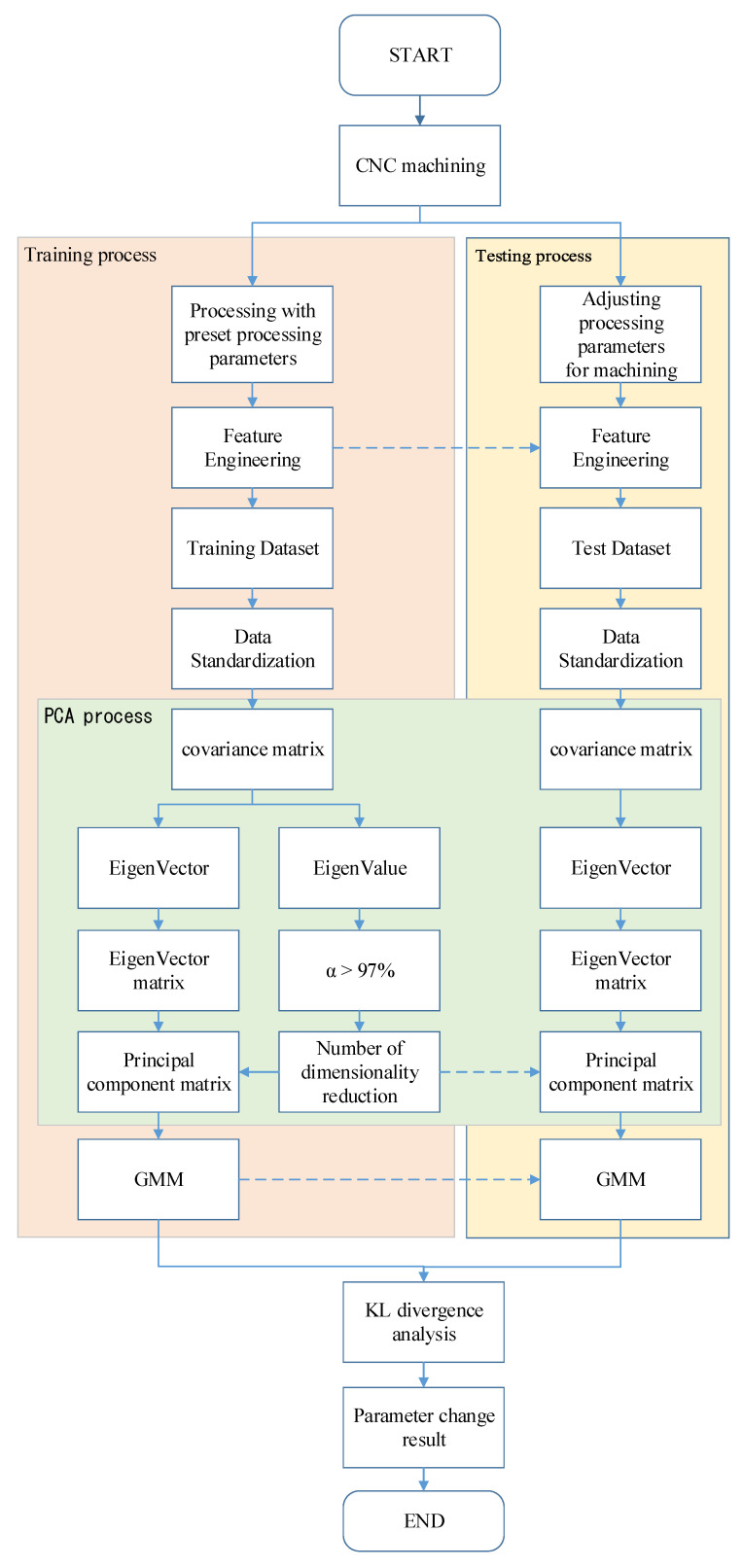
Experimental flowchart for GMM diagnosis.

The experimental flowchart is shown in Figure 9. A three-axis accelerator attached to the workpiece and a current sensor clamped with an inverter module of the spindle were all sampled with 12.8 kHz. There were about 4 min and a few seconds for the acquisitioned time when machining an aluminum workpiece. A total of 30 workpieces were experimentally machined. There were two datasets for each machined workpiece. The first dataset was collected for the initial cutting based on a spindle speed of 6000 rpm. These data corresponded to consecutive sensing signals, which were divided starting from the first data number section up to the 65th data number section during machining. The second dataset was collected but with a change in the spindle speed to 5500 rpm. This dataset corresponded to the sensing signals divided from the 66th data number section to the 129th data number sections. Since every section was with 2 s, a total of 129 data numbers would be analyzed by the feature engineering first, followed by PCA for dimensional reduction and then unsupervised learning via the GMM. All the features’ dimensions were reduced based on the PCA with a cumulative contribution ratio, α, more than 97%. Finally, the GMM was applied as the diagnostic method with the measure of the Kullback–Leibler divergence (KLD). Then, a threshold design value can be assigned by diagnosing the spindle speed change of a CNC machine tool during milling.

## 4. Results and Discussion

### 4.1. Feature Engineering, PCA, and F-Score

The core concept of feature engineering (FE) is to identify the most discriminative features from raw data by using a systematic operation. The fusing of different sensor signal features is discussed prominently in recent research. Those signals are obtained from different sensors when it is unclear which one is the key sensing signal for the characteristics of the object requiring diagnosis. In this study, data segmentation was conducted first. The raw data were divided into 129 sections (within 4 min and few seconds) with a constant of 2 s to acquire the signals of the part by filtering the data value with six standard deviations. Secondly, feature extraction was implemented. Six statistical features, namely, root mean square (RMS), kurtosis, skewness, crest factor, variance, and standard deviation, were extracted in each section of the time domain. Four statistical features, namely, mean, standard deviation, skewness, and kurtosis, were extracted in each section of Power Spectrum Density (PSD)-Amp and PSD-Shape. Therefore, eight PSD features were made. For the purpose of more feature argumentation, the vibrational and current sensing data were also transformed to the Fast Fourier Transform with a Nyquist frequency of 6.4 kHz. The average amplitude of each frequency sector was calculated every 400 Hz. In Table 4, 46 (6 + 8 + 32) features acquired from vibrational and current features with 25,600 data (2 s) in one segment were made. Each segment was divided into 128 sections. Therefore, 200 identities with each 46 features were constituted for dimensional reduction via PCA. Reduced feature dimensions of PCA were calculated with a cumulative contribution ratio more than 97%. In Table 5, the dimensionality was reduced from 46 features to 23.

### 4.2. GMM Modeling and Visualization

In this study, both online and offline spindle speed variable monitoring procedures were used. The offline monitoring was performed using a program written in MATLAB. The online monitoring program for the GMM and PCA was achieved using a C# program. The processed raw data by feature engineering were fed through MATLAB and treated as dynamic-linked libraries. Then, these features were included in the real-time C# programming. The synergized FE-PCA-GMM/KLD was constructed thereof. A man-and-machine interface (MMI) can be built for industrial practice.

**Figure 10:**
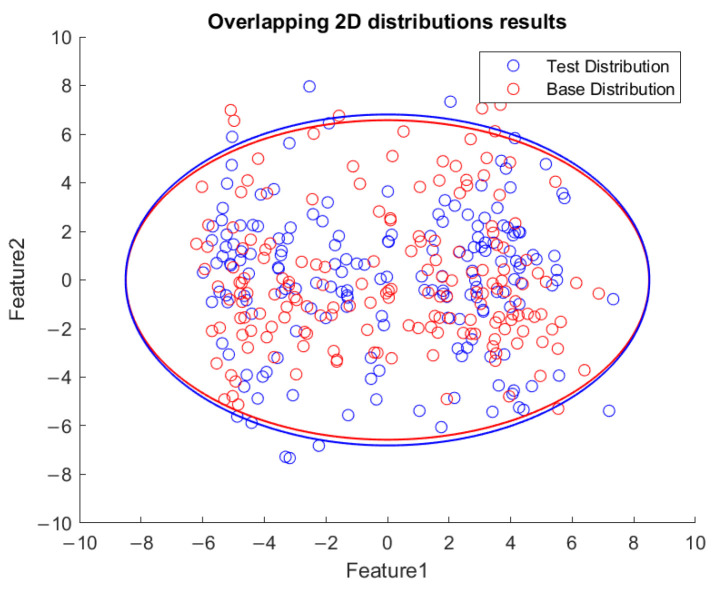
Feature distribution along the *x*-axis, 6000 RPM.

**Figure 11:**
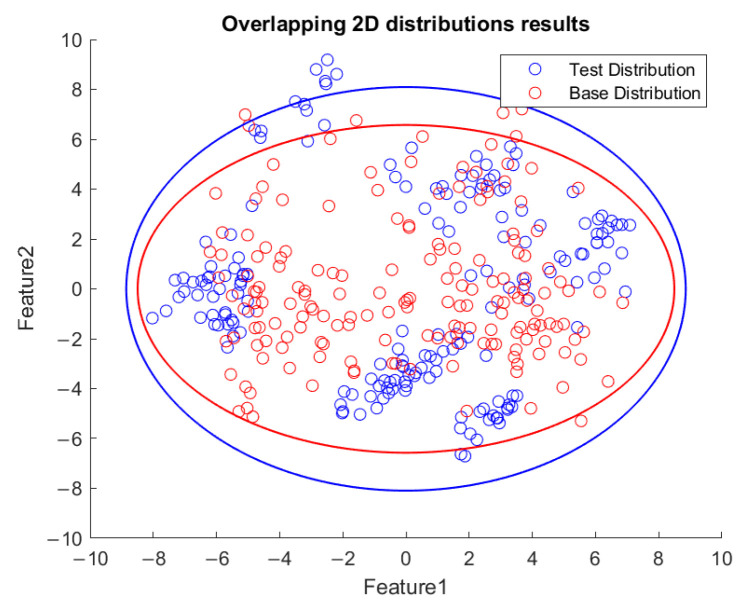
Feature distribution along the *x*-axis, 5500 RPM.

**Figure 12:**
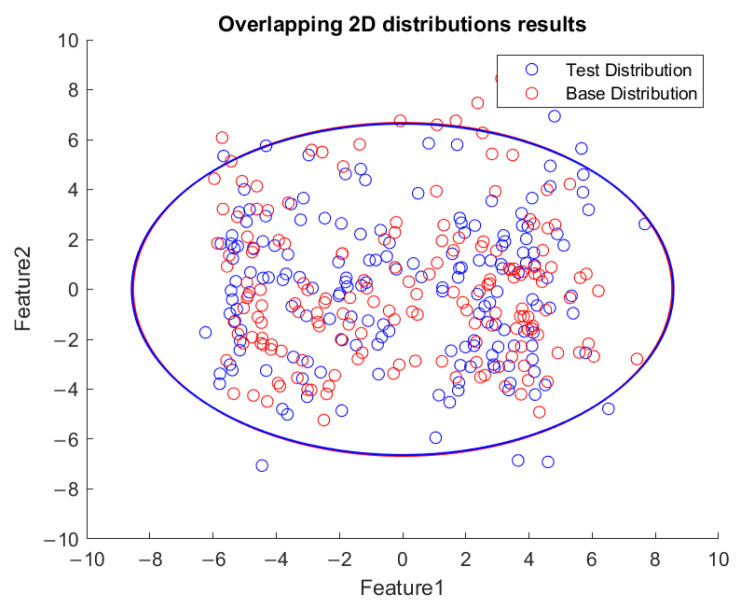
Feature distribution along *y*-axis, 6000 RPM.

**Figure 13:**
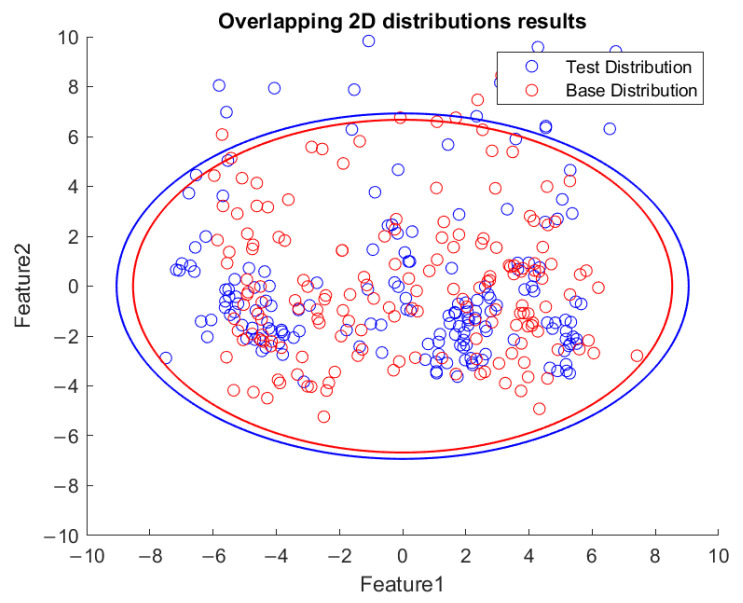
Feature distribution along the *y*-axis, 5500 RPM.

**Figure 14:**
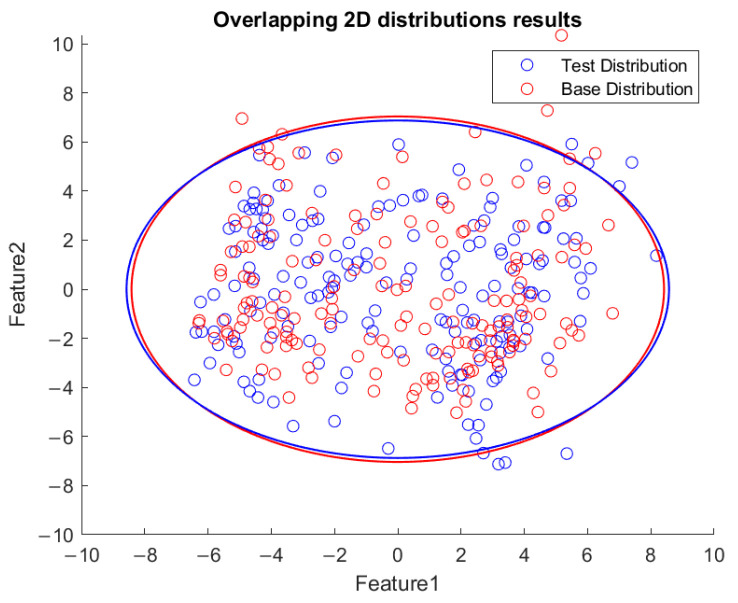
Feature distribution along the *z*-axis, 6000 RPM.

**Figure 15:**
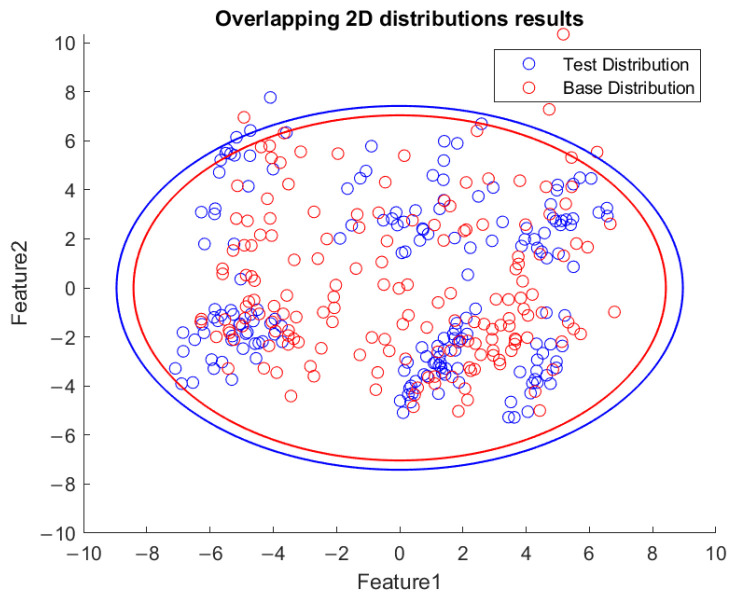
Feature distribution along the *z*-axis, 5500 RPM.

Based on the flowchart in Figure 9, Figure 10 demonstrates the visualized plot of spindle speed of 6000 rpm, by using the top two features of the *x*-axis acceleration for the FE-PCA-GMM method. The red circles are the training data set while the blue ones are the test dataset. Without adjusting the spindle speed (the top right block diagram in Figure 9), we can see that the blue circles were pretty close to the red circles. Based on the same flowchart in Figure 9, Feature 11 demonstrates the plot with the test spindle speed changed to 5500 rpm. The red circles are the same training dataset, while the blue ones are the test dataset. We can see that the distribution of blue circles was not as the same as that of the red circles. Such visualized plotted depictions deformed with larger elliptical shape. This coincides with the change in $\mu_{i}$ and $\Sigma_{i}$ by the characteristics of GMM. Under the same experimental results of Figure 10 and Figure 11, Figure 12 and Figure 13 show the results for the *y*-axis and Figure 14 and Figure 15 show the results for the *z*-axis. These plots showed the same distribution of blue circles, which was the same as the red ones when the spindle’s speed was the same. The blue contour deviated from the red contour when the spindle’s speed was changed. These visualized diagnosed plots demonstrate that the developed FE-PCA-GMM can perform speed change diagnosis with workpiece machining.

### 4.3. KLD and KLD’s Five-Point Moving Average

As mentioned, the 1–65th data section was for the spindle speed of 6000 rpm, while the 65th–129th data section was changed to 5500 rpm. These data corresponded to the first to fourth cutting paths, followed by the 5th to 8th cutting paths, as shown in Figure 7. In this experiment, the threshold value of KLD was set as 1. The definition for the confusion matrix based on the value of KLD is shown in Table 6. TP, TN, FP, and FN indicate the true positive, true negative, false positive, and false negative, respectively. Table 7 shows the values of the confusion matrix for each axis. The calculated F-Score using Equation (17) for each axis is demonstrated in Table 7. The F1-scores for X, Y, and Z axes are approximately 0.95, 0.88, and 0.93, respectively. Since the F1-score is one of the common statistics besides accuracy, the larger the F-score is, the better the comprehensive performance of the model becomes [28]. The F-measure in *x*-axis clustering is high when compared with the *z*-axis and *y*-axis. The experimental results indicate that by assigning the threshold of the KLD appropriately, the FE-PCA-GMM model can predict the spindle change from 6000 rpm to the lower speed of 5500 rpm successfully.

**Figure 16:**
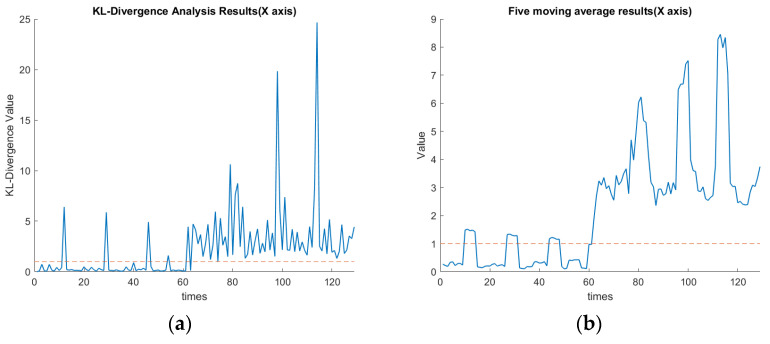
(**a**) KLD of rotational speed change along the *x*-axis (seconds). (**b**) Five-point moving average of Figure 16a along the *x*-axis (seconds).

**Figure 17:**
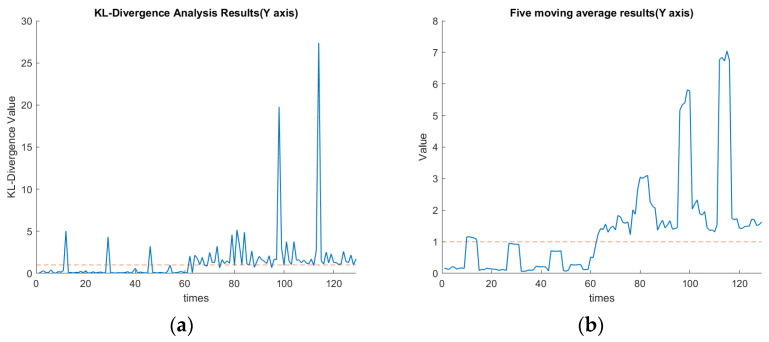
(**a**) KLD of rotational speed changes along the *y*-axis (seconds). (**b**) Five-point moving average of Figure 17a along the *y*-axis (seconds).

**Figure 18:**
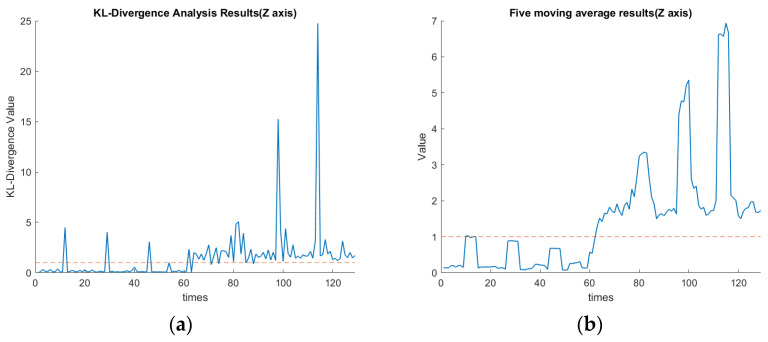
(**a**) KLD of rotational speed changes along the *z*-axis (seconds). (**b**) Five-point moving average of Figure 18a along the *z*-axis (seconds).

Figure 16a,b show the plots of the KLD of the rotational speed change along the *x*-axis. These two figures indicate the average KLD values for milling the workpiece by a cutting depth of 1 mm eight times when the cutting process moves to the next milling surface. The orange dashed line in Figure 16, Figure 17 and Figure 18 indicates the assigned threshold value. Table 8 details the average KLD values for each axis with and without data cleaning. The purpose of performing the data cleaning was because the acquired data of the tool vibration were too small or even close to zero when the tool was left out of the workpiece and then proceeded to perform the next milling process. As we can see in the 12th, 29th, and 46th rounds of Figure 16a, the surge in KLD value was due to the GMM’s calculation, since there were weak vibrational signals when the spindle cutting tool was not in contact with the workpiece. Such a scenario enlarged the value of the KLD since the calculated mean and covariance of the GMM during the uncontacted cutting process deviated significantly. This will cause a significant variation in the KLDs and then over the preassigned threshold. This will issue a warning signal in practice. Nevertheless, as we can see in the 80th, 97th, and 114th rounds of Figure 16a, the KLD value remained higher than the threshold since the spindle’s speed was changed and then favored the deviation in the calculated mean and covariance of the GMM’s thereof. Comparing Figure 17a with 17b in the *y*-axis and Figure 18a with 18b in the *z*-axis, the diagnosis of using GMM/KLD illustrated more robustness by the five-point moving average when the spindle speed was reduced to 5500 rpm. The result of FN in Table 7 for each axis should be caused by no data cleaning in the real-time C # program. As shown in Table 8, the use of data cleaning could relieve the possibility of warning signals in practice. To conclude here, the use of FE-PCA-GMM/KLD preserved the improved prognostic method for the spindle’s speed change of the CNC machine tool.

**Figure 19:**
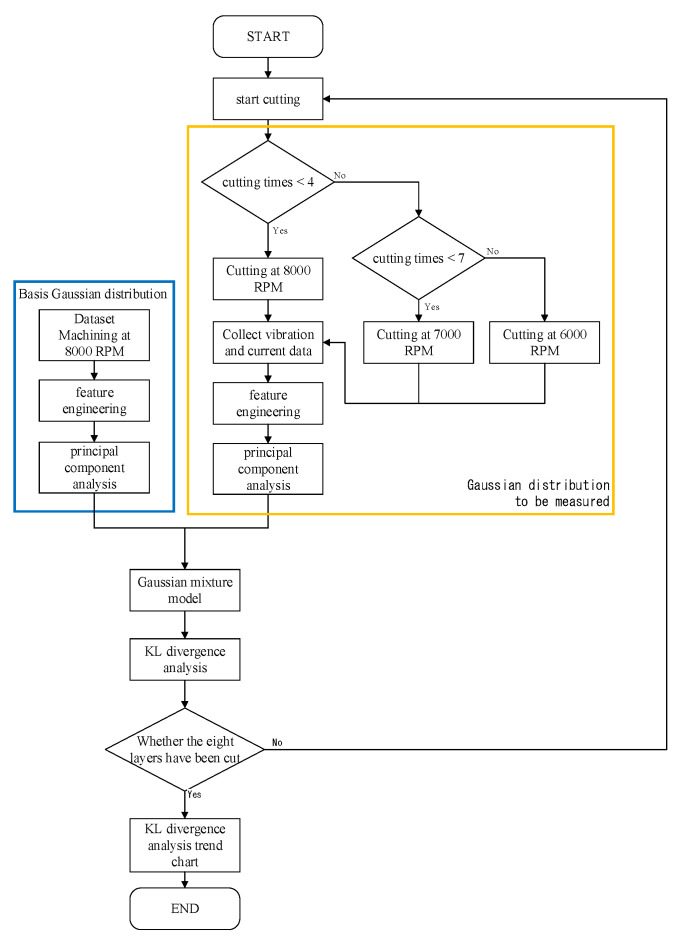
Experimental flowchart for continuous diagnosis of GMM with the speed from 8000 RPM down to 6000 RPM.

**Figure 20:**
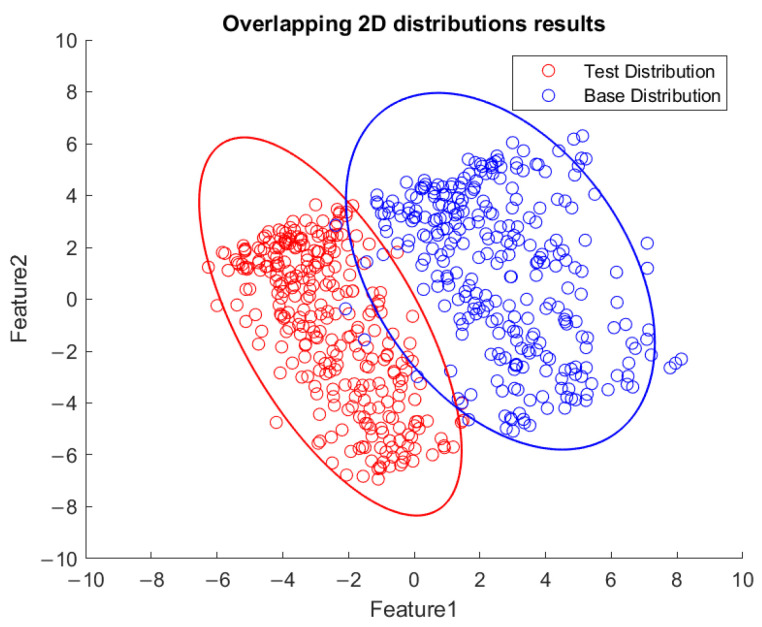
Visualized GMM two-feature distribution along the *x*-axis for 8000 rpm.

**Figure 21:**
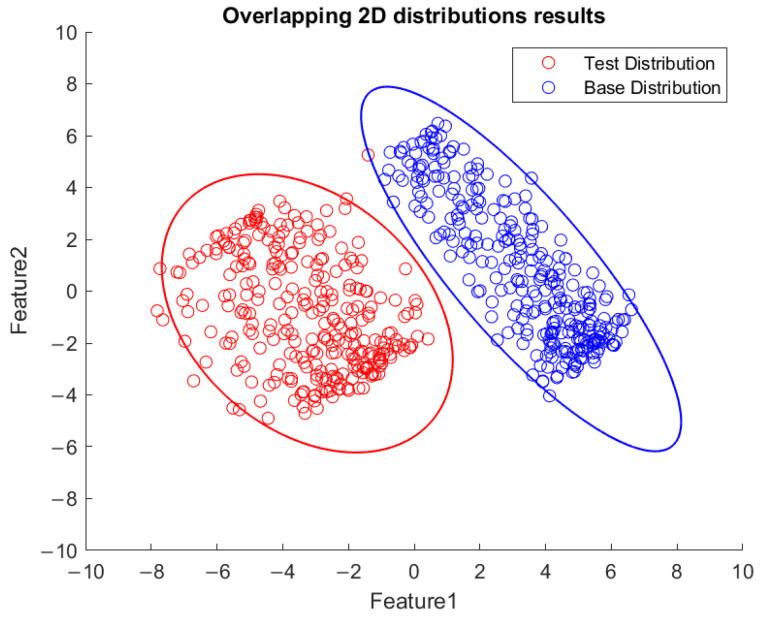
Visualized GMM two-feature distribution along the *x*-axis for speed of 8000 RPM down to 7000 RPM.

**Figure 22:**
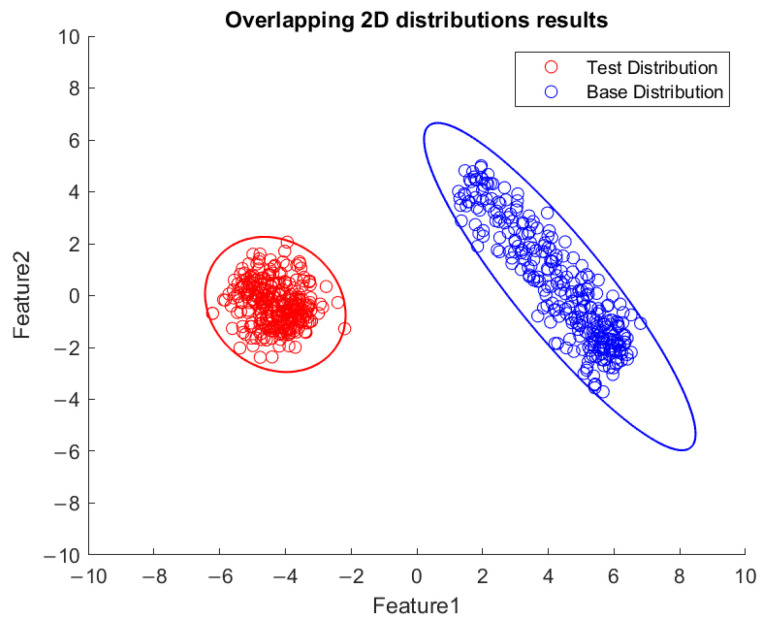
Visualized GMM two-feature distribution along the *x*-axis for speed of 8000 RPM down to 6000 RPM.

**Figure 23:**
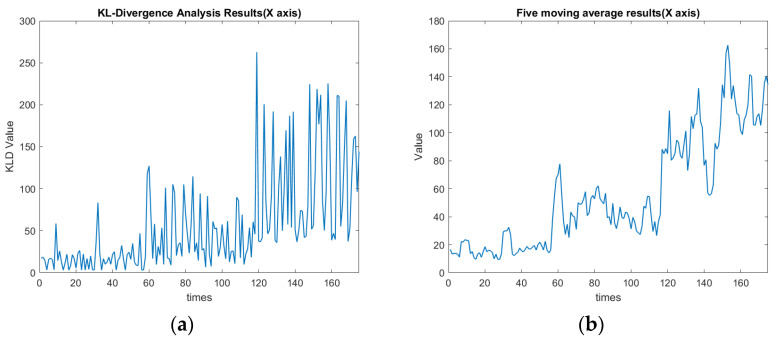
(**a**) KLD value of speed changes from 8000 rpm down to 7000 rpm and then to 6000 rpm along the *x*-axis (seconds). (**b**) Five-point moving-average of Figure 18a along the *x*-axis (seconds).

Thus, an example was tested by changing the spindle speed from 8000 rpm to 7000 rpm and then down to 6000 rpm. The consecutive FE-PCA-GMM/KLD program was implemented as shown in Figure 19. The unsupervised learning of the GMM from the initial 8000 rpm to 7000 rpm and finally to 6000 rpm was conducted and the plots of the top two features are shown in Figure 20, Figure 21 and Figure 22, respectively. This demonstrated the phenomena of the more visualized separation when the speed difference was enlarged. As estimated, the value of KLD increased when the spindle speed decreased from 8000 rpm to 7000 rpm and then down to 6000 during the machining process in Figure 23. The experimental results show affirmation toward the proposed method concerning variable speed changes.

## 5. Conclusions

In this research, the effectiveness of the developed method for determining the manufacturing parameter changes of a CNC machine tool is verified by experiments. The vibrations and spindle current data gathered with an accelerometer attached to a vise and current sensor clamped with spindle current, respectively, were used to produce a dataset by increasing their dimensionality with feature engineering and then reducing the dimensionality with PCA. Then, the GMM with the KLD measure performed the prognostic diagnosis in real-time. The top two features’ visualized results and an F1 score more than 0.88 revealed that the overlap between any two Gaussian mixed distributions was higher with CNC machine tooling with a preset spindle speed of 6000 RPM, suggesting greater similarity (lower when the rotational speed was reduced to 5500 RPM (less similarity)). In addition, the visualized results of the two features reveal that the clustering behavior in between the Gaussian mixed distributions was significantly distinct when the CNC machine tooling with a spindle speed changed from 80,000 RPM to 6000 RPM. Finally, with the merit of high sensitivity by the KLD, the trend of the KLD changed considerably when the machine spindle speed was changed. The established unsupervised FE-PCA-GMM/KLD method can be applied to issue warnings when it predicts a manufacturing process parameter change.

The present research will continue to be implemented for other machining parameter changes. The in situ C # program for GMM-KLD will be improved with automatic data cleaning via the G-code when the spindle tool is left out of the cutting surface. Object detection will be conducted to capture the GMM’s morphing in a future study. In conclusion, according to the results of this paper, there is potential to develop a smart device and deploy an unsupervised FE-PCA-GMM/KLD system. A smart manufacturing method that automatically performs the prognostic diagnosis for the CNC machine tools can be achieved.

## Figures and Tables

**Table 1:** DMARK-180P specifications.

Spindle	Speed	24,000 rpm (max), 2.2 kW
	Motor	Built-in
	Cooling	Cold air
Servo motor	*x*-axis	400 W
	*y*-axis	400 W
	*z*-axis	400 W
Servo drive	A2, 400 W, single-phase/three-phase connections	
Travel	*x*-axis	160 mm
	*y*-axis	180 mm
	*z*-axis	150 mm
Feed	Rapid movement (G0)	Maximum *x*-,*y*-, and *z*-axis speeds: 6/6/6 m/min
	Control precision (controller)	0.001 mm
Machine body	Length	750 mm
	Width	500 mm
	Height	1400 mm
Control	Control format	Standard G code, standard M code
	Connection modes	ETHERNET/USB

**Table 2:** BALTech DAQ-204 specifications.

Input channels	2/4 channels
IEPE	±10 V
Maximum sampling frequency	102,400 Hz
Bandwidth range	12.8 kHz

**Table 3:** Triaxial accelerometer specifications.

Model	BT-1513
Acceleration range	±50 g
Bandwidth	0.5–15 kHz
Triaxial sensitivity	X: 108.85 mV/g
	Y: 100.48 mV/g
	Z: 103.64 mV/g

**Table 4:** All 46 features for every data segment.

Total Data of 25,600 (Vibration Value) + 25,600 (Current Value) in One Data Segment		
Order	Feature	Sensing Data
1	RMS	vibration value
2	Kurtosis	
3	Variance	
4	Crest Factor	
5	Standard deviation	
6	Skewness	
7–22	Average amplitude of frequency sectors [1 + 400 × (i − 1)] Hz~(400 × i) Hz, i = 1,2,3,…,16	
23–38	Average amplitude of frequency sectors [1 + 400 × (i − 1)] Hz~(400 × i) Hz, i = 1,2,3,…,16	current value
39	PSD-Amplitude Mean	vibration value
40	PSD-Amplitude Standard deviation	
41	PSD-Amplitude Skewness	
42	PSD-Amplitude Kurtosis	
43	PSD-Shape Mean	
44	PSD-Shape Standard deviation	
45	PSD-Shape Skewness	
46	PSD-Shape Kurtosis	

**Table 5:** PCA dimensionality before and after reduction.

		Before	After
	
*x*-axis		200 × 46	200 × 23
*y*-axis		200 × 46	200 × 23
*z*-axis		200 × 46	200 × 23

**Table 6:** Definition for the confusion matrix based on the value of KLD.

	KLD < 1	KLD > 1
KLD < 1 (1–65th data section tests: 6000 rpm)	TP	FN
KLD > 1 (65th–129th data section tests: 5500 rpm)	FP	TN

**Table 7:** The values of the confusion matrix and the F-Score for each axis.

	TP	FN	FP	TN	F-Score
*x*-axis	58	6	0	65	0.9508
*y*-axis	59	5	10	55	0.8872
*z*-axis	59	5	4	61	0.9291

**Table 8:** The average KLD values for each axis with and without data cleaning when the cutting process moves to the next milling surface.

	6000 RPM (1–65th Data Section) without Data Cleaning	6000 RPM (1–65th Data Section) with Data Cleaning	5500 RPM (65th–129th Data Section) without Data Cleaning	5500 RPM (65th–129th Data Section) with Data Cleaning
*x*-axis	0.675	0.43	3.953	3.244
*y*-axis	0.409	0.226	2.448	1.767
*z*-axis	0.404	0.237	2.562	1.9

## Data Availability

Not available.
